# MicroRNAs as theranostic markers in cardiac allograft transplantation: from murine models to clinical practice

**DOI:** 10.7150/thno.56327

**Published:** 2021-04-07

**Authors:** Jan Novák, Táňa Macháčková, Jan Krejčí, Julie Bienertová-Vašků, Ondřej Slabý

**Affiliations:** 1 Department of Pathological Physiology, Faculty of Medicine, Masaryk University, Kamenice 5-A18, 625 00, Brno, Czech Republic.; 2 Second Department of Internal Medicine, St. Anne's University Hospital and Faculty of Medicine, Masaryk University, Pekařská 53, 65691, Brno, Czech Republic.; 3 Central European Institute of Technology, Masaryk University, Kamenice 5-A35, 625 00, Brno, Czech Republic.; 4 Department of Cardiovascular Diseases, St. Anne's University Hospital and Faculty of Medicine, Masaryk University, Pekařská 53, 65691, Brno, Czech Republic.; 5 RECETOX, Faculty of Sciences, Masaryk University, Kamenice 5-A29, 625 00, Brno, Czech Republic.; 6 Department of Biology, Faculty of Medicine, Masaryk University, Kamenice 5, 625 00, Brno, Czech Republic.

**Keywords:** microRNA, biomarker, cardiac allograft transplantation, acute cellular rejection, vasculopathy

## Abstract

Congestive heart failure affects about 23 million people worldwide, and cardiac allograft transplantation remains one of the last options for patients with terminal refractory heart failure. Besides the infectious or oncological complications, the prognosis of patients after heart transplantation is affected by acute cellular or antibody-mediated rejection and allograft vasculopathy development. Current monitoring of both conditions requires the performance of invasive procedures (endomyocardial biopsy sampling and coronary angiography or optical coherence tomography, respectively) that are costly, time-demanding, and non-comfortable for the patient.

Within this narrative review, we focus on the potential pathophysiological and clinical roles of microRNAs (miRNAs, miRs) in the field of cardiac allograft transplantation. Firstly, we provide a general introduction about the status of cardiac allograft function monitoring and the discovery of miRNAs as post-transcriptional regulators of gene expression and clinically relevant biomarkers found in the extracellular fluid. After this general introduction, information from animal and human studies are summarized to underline the importance of miRNAs both in the pathophysiology of the rejection process, the possibility of its modulation by altering miRNAs levels, and last but not least, about the use of miRNAs in the clinical practice to diagnose or predict the rejection occurrence.

## Cardiac allograft transplantation and rejection monitoring - status

Congestive heart failure affects about 23 million people worldwide, and affected individuals present to the clinics with the various signs of cardiac insufficiency - among others, we can list dyspnoea, swellings, and intolerance of exercise 1,2. Cardiac allograft transplantation (CAT) remains one of the last options for patients with terminal refractory heart failure in whose the maximal intensive pharmacological and device therapy does not lead to the relief of congestive symptoms and who still reside in the functional class IV of New York Heart Association (NYHA) classification 2,3. After heart transplantation, the prognosis of patients is affected by numerous events, with one of the most serious being graft rejection, especially during the first months after transplantation 4. The most commonly seen type of rejection is acute cellular rejection (ACR), followed by antibody-mediated rejection (AMR, also called non-cellular or vascular rejection) or mixed rejection (i.e., the occurrence of both ACR and AMR at the same time) 5. ACR represents a T-cell mediated damage to the cardiac allograft characterized by cellular infiltration of the myocardium. In contrast, AMR represents antibody-mediated damage to the cardiac allograft, characterized by the presence of specific antibodies created by B-cells identified in the myocardium 6. Each rejection type represents a particular entity and needs to be addressed accordingly by choosing the appropriate immunosuppressive therapy regimen 7,8. While the incidence of higher grades of ACR significantly decreased after the introduction of novel immunosuppressive reagents (from 54% in 1990 to 5% in 2020 according to Subehrwal et al. or more recently from 30% in between 2004-2006 to 12% in between 2017 and 2018 according to ISHLT report 9, the prevalence of AMR and MR remains approximately the same (about 20% of patients in the long term 9,10).

In the later months and during the years after CAT, the risk of ACR and AMR decreases, and the patients are jeopardized by the development of cardiac allograft vasculopathy (CAV) that is defined by the development of concentric fibromuscular intimal hyperplasia lesions within epicardial and intramyocardial arteries and by the development of eccentric atherosclerotic plaques in the epicardial arteries 11. Incidence of CAV differs among published studies ranging from 18% to 54.8%, based on the methodology (the coronary angiography showing lower sensitivity compared to intravascular ultrasound or OCT) and the time from CAT (with the higher prevalence of CAV in patients longer from the operation) 12-14. Further threats that a clinician that takes care of the patients after CAT needs to be aware of include, e.g., infectious or oncological complications primarily associated with the immunosuppressive treatment. However, these complications are not the focus of the current review that focuses on graft rejection's pathophysiology and monitoring.

Currently, the prevention of graft rejection and general surveillance of the transplanted patients includes the set of regular follow-ups that involve clinical and echocardiographic examination and crucially the performance of endomyocardial biopsy (EMB) with the subsequent histopathologic evaluation of biopsy samples using the International Society of Heart and Lung Transplantation (ISHLT) grading systems 4,15,16. Neither regularity nor number of these follow-ups is determined in any clinical trial. It is always necessary to balance the clinical utility of biopsy and its uncommon but serious complications, among others, e.g., ventricular fibrillation or cardiac tamponade 4,15. Nevertheless, this standard set of EMBs shall guide clinicians in setting up the appropriate dosing of immunosuppressant therapy, which must be neither too low to avoid rejections nor too high to prevent adverse effects related to profound immunosuppression.

Currently used histopathologic evaluation of EMB recognizes graft rejection at its histological level, i.e., when the tissue is already infiltrated with immune cells and its structure is altered. Such changes shall result in adequate changes in the immunosuppressive therapy to avoid acute rejection's clinical manifestation. According to the time-line model of organ rejection, before the changes on the clinical and histological level, changes also occur on the biochemical and molecular level, and identification of biochemical and molecular markers may thus lead to the earlier detection of graft rejection or even prevent its occurrence on the histological level 17. As the event of any acute rejection is predictive for further developing chronic rejection, the prevention of any single rejection episode shall improve the graft survival and function and, consequently, patients' quality of life 18,19.

Since the programs of heart transplantation started, various non-invasive approaches and biomarkers were tested for acute rejection monitoring, including imaging methods (ECG, echocardiography, magnetic resonance imaging, gallium scintigraphy) 20, immunological status monitoring (various interleukin or interleukin receptors levels, flow cytometry, and cytoimmunologic monitoring) or biochemical markers (NT-proBNP, troponin, prolactin, urinary polyamines, etc.) 21,22. In recent years use of AlloMap, i.e., gene expression profiling of circulating leukocytes, has been initiated in some institutions. It was shown to be useful for rejection monitoring and partially reduced the number of performer routine biopsies 23. However, even this method does not present sufficient specificity and sensitivity to replace EMB fully. Thus, the performance of EMB remains the gold standard in the current follow-up protocols, and the search for novel biomarkers continues.

With the progress in molecular biology and genetics, especially with the development and improvement of nucleic acid isolation techniques and next-generation sequencing, a novel group of molecular biomarkers based on circulating nucleic acids has emerged. Both circulating deoxyribonucleic (DNA) 24 or ribonucleic acids (RNA) may be detected in bodily fluids of transplanted patients. Specifically, microRNAs (miRNAs, miRs) seem to represent the promising biomarkers group not only in the field of cardiac transplantation but non-invasive diagnostics in general 25,26.

This narrative review first provides a brief insight into the transcriptomics-derived biomarkers discovery focusing on miRNAs biology and functions. We then summarize the current knowledge about the role of miRNAs in heart transplantation, firstly focusing on their pathophysiological role studied in murine models of CAT and secondly focusing on their potential use in clinical practice as biomarkers as described in human studies.

## MicroRNAs as a Class of Non-coding RNAs

Within the completion of the HUGO 27 and ENCODE 28 projects, it became apparent that about 85% of the genome is actively transcribed (called “transcriptome”), and only 2-3% of the transcriptome (known as protein-coding RNAs) are being translated into proteins. The remaining massive group of RNAs became known as the non-coding RNAs (ncRNAs). It has been repeatedly shown that they possess crucial regulatory functions in the gene expression process, being involved in all thinkable processes in the human body, including but not limited to cellular growth, apoptosis, fibrosis, hypertrophy, or aging 29,30. Based on their length, they can be divided into two main groups, small non-coding RNAs (sncRNAs) and long non-coding RNAs (lncRNAs), with the size of 200 base pairs being determined as an arbitrary border.

Another way how ncRNAs can be classified is the division into structural and regulatory RNAs 30. Structural RNAs comprise several classes of sncRNAs (ribosomal, transfer, small nuclear, and small nucleolar RNAs). In contrast, lncRNAs and other sncRNAs classes (miRNAs, PIWI-interacting RNAs [piRNAs], and small interfering RNAs [siRNAs]) are considered as regulatory RNAs 30. Structural RNAs are more prone to be consistently expressed. In contrast, levels of regulatory RNAs changes under various circumstances and differ in the ordinary and diseased states of the specific tissues, i.e., their expression is altered in the presence of disease, which makes them potential biomarkers.

**Figure 1:**
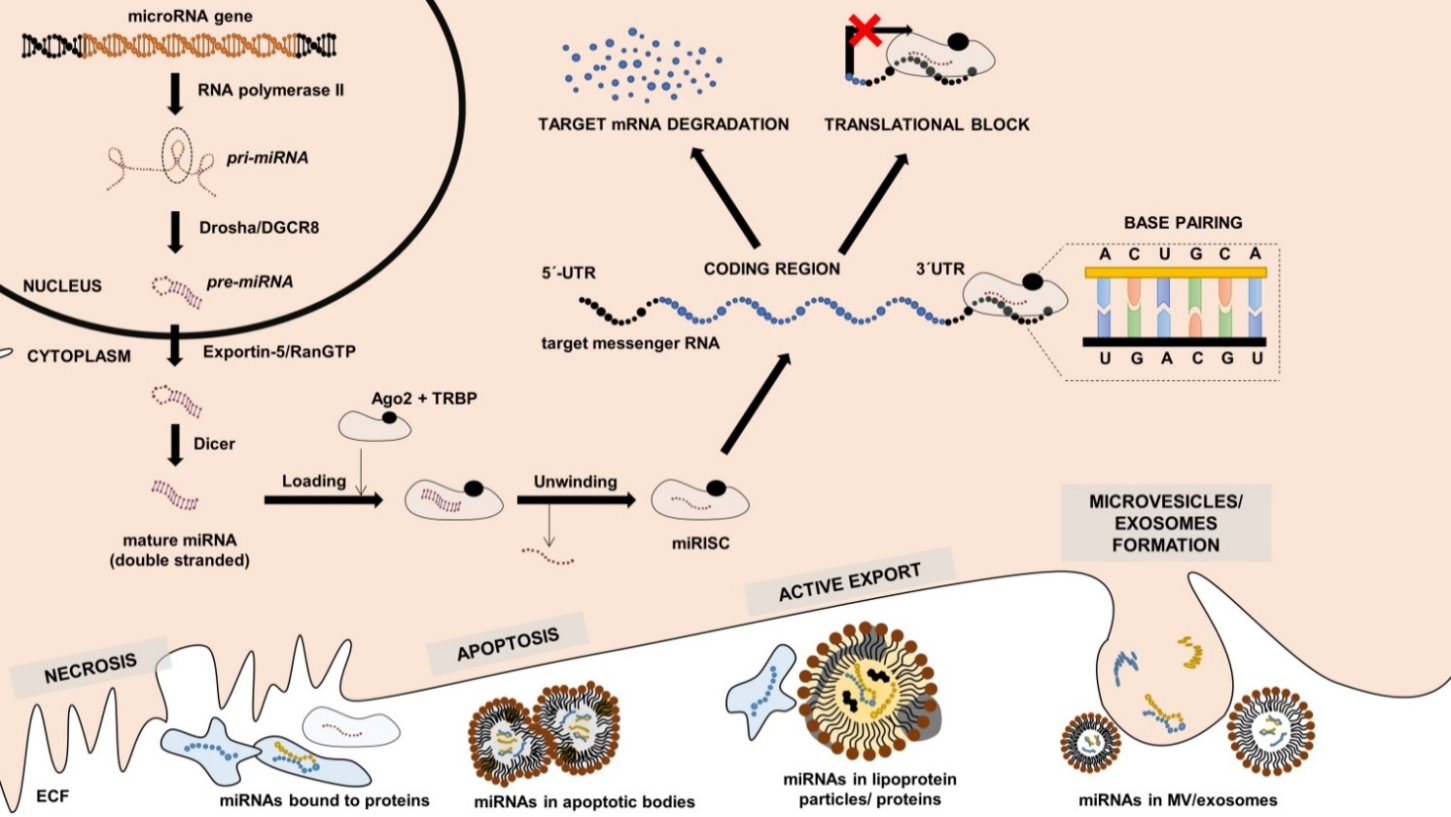
** Canonical miRNA expression pathway, miRNA function and release into the extracellular fluid.** miRNA gene is transcribed by RNA-polymerase II into the primary miRNA (pri-miRNA) transcript, which is cleaved by Drosha/DGCR8 to form precursor miRNA (pre-miRNA) with typical hairpin structure. pre-miRNA exported from the nucleus by Exportin-5/RANGTP into the cytoplasm, where it is cleaved by Dicer in mature double-stranded miRNA. Mature is loaded into the complex with Argonaut (Ago) proteins, unwinded, and the final functional unit is called microRNA-induces silencing complex (miRISC). mRISC targets messenger RNAs (mRNAs) based on base-pair complementarity, and its binding lead either to target mRNA degradation or translational inhibition. Intracellular miRNAs can be transferred into the extracellular space either by cell necrosis, apoptosis, active export, or by the formation of microvesicles, exosomes. References are provided within the main text.

miRNAs represent a highly conserved group of molecules among the living organisms that mainly regulate gene expression on the post-transcriptional level either by blocking mRNA translation to proteins or by promoting RNA decay (or by combing both mechanisms) 31. There are several ways how miRNAs can be generated; however, the most common canonical pathway starts when miRNA genes are transcribed by RNA polymerase II into primary miRNA (pri-miRNA) transcripts (Figure 1). These transcripts are then processed by microprocessor complex (Drosha/DGCR8) already in the nucleus into the precursor miRNAs (pre-miRNAs) with typical hairpin structure. pre-miRNAs are then transferred into the cytoplasm. There they are cleaved by endonuclease Dicer into double-stranded miRNAs, and one of the strands is then loaded into the Argonaut (Ago) protein complex creating a miRNA-induced silencing complex (miRISC). Based on base pairs complementarity, miRNA molecule in the miRISC then guides the complex to the complementary region (typically located in the 3'untranslated region) of the targeted mRNA strands, and binding of miRISC results in lowering of the levels of protein encoded by target mRNA 32. Some of the miRNAs are expressed ubiquitously, while the others are tissue-specific, i.e., being expressed 20-40 times more in the individual tissues 33. Furthermore, some of the miRNAs seem to act more as “fine tuners”, i.e., they are functionally abundant and may be replaced by other miRNAs, while some of them seem to play critical roles in regulating gene expression in the specific tissue, and their absence results in developmental and functional defects 31,32.

Considering their essential roles in regulating gene expression, determining the miRNA expression in tissues may provide valuable physiologically and, more importantly, also clinically relevant information about the disease's current status or future course - as repeatedly referred in various malignancies 25,34. However, the widespread use of *tissue* miRNA expression in cardiology is limited by the lack of easily accessible myocardial samples. Intriguingly, besides important intracellular roles of miRNAs in individual tissues, it is becoming evident that miRNAs are also released into the extracellular fluid, including plasma, urine, or even saliva 35, that are routinely used in the current clinical practice.

miRNAs can be transferred to extracellular space by various mechanisms: they can be actively secreted from cells (e.g., by neutral sphingomyelinase 2 36), they can be loaded into the extracellular vesicles or lipoprotein molecules 37,38, or they can be released during the cellular death, including both necrosis or apoptosis 39. Despite the high extracellular RNase activity, miRNAs in extracellular space are protected from cleavage by binding to specific proteins (e.g., Ago proteins) or by being packed in microvesicles, apoptotic bodies, or lipoprotein particles 40,41. miRNA levels are stable among the individuals from the same species. They are resistant to repeated freeze-thaw cycles, and their levels are not decreasing even after more than 15 years of storage in ultra-low temperatures 42. Their levels in the extracellular fluids are not stochastic and determining them may therefore reflect changes occurring within the organism and provide diagnostic or prognostic information about the individual patient. Listed properties are common for all small non-coding RNAs, which makes them promising ideal biomarkers. However, there is no data about other sncRNAs classes than miRNAs in the field of CAT. Compared to the lncRNAs, all sncRNAs are more stable in biological samples and are more easily technically accessible 43.

Several methods and approaches may be employed when searching for novel clinically relevant miRNA species in the specific disease. Firstly, high-throughput methods including either small RNA sequencing or microarray profiling may be used 44. These methods detect a wide range of miRNAs in individual samples, and in the case of small RNA sequencing also allows for isomiR and novel variants detection. By comparing miRNA profiles from control and diseased samples, relevant miRNAs with biomarker potential are then identified. However, strict rules need to be applied for a further selection of relevant miRNAs, and results from RNA sequencing always need to be validated on independent cohorts. For validation, qRT-PCR based methods represent the gold standard, and potentially newly developed Mireia procedures (that are similar to currently widely used ELISA procedures) will be used in the near future 45. Secondly, besides the high-throughput approaches, bioinformatics *in silico* prediction of relevant miRNAs altered in the specific disease may be performed by a screening of currently accessible databases (Table 1), and lastly, performing literature screening may help to identify relevant miRNAs that will be altered in the specific disease. All of these methods have their advantages and limitations, and usually, the combination of more of them yields the most relevant results.

## Animal studies focusing on heart transplantation

### Murine models of heart transplantation - general terms

#### Xenotransplantation and allotransplantation

When studying physiology and pathophysiology related to cardiac transplantation, either xenotransplantation or allotransplantation is performed. Heart xenotransplantation represents the heart transplantation between two different species, e.g., between rat and mouse. In contrast, heart allotransplantation is defined as the transplantation of the heart between the same species' individuals that are not genetically identical, e.g., between two different mouse strains. Most of the studies cited further performs cardiac allotransplantation (that is currently the only option for heart transplantation in human), most commonly between C57BL/6 and BALB/C mouse strains.

General study designs involve a *control (syngeneic) group* of animals whose cardiac transplantation is performed within one strain (typically from C57BL/6 to C57BL/6 mice), while graft rejections are studied in the *rejection (allogeneic) group* where transplantation between two strains (from BALC/C to C57BL/6 36,37 or vice versa 38,39) is performed. Such study design ensures that both animals in the *control* and *rejection* groups are operated and that observed changes in miRNA expression are not related just to ischemia-reperfusion (I/R) injury. Previously mentioned mouse strains represent genetically engineered animals that present with immunologically divergent responses of macrophages and T cells 46. In C57BL/6 mice, Th1 predominant and inflammatory macrophages (M1) responses are observed, while in Balb/c mice, Th2 predominant and wound healing macrophage (M2) reactions are present 46,47.

#### Orthotopic and heterotopic heart transplantation

**Figure 2:**
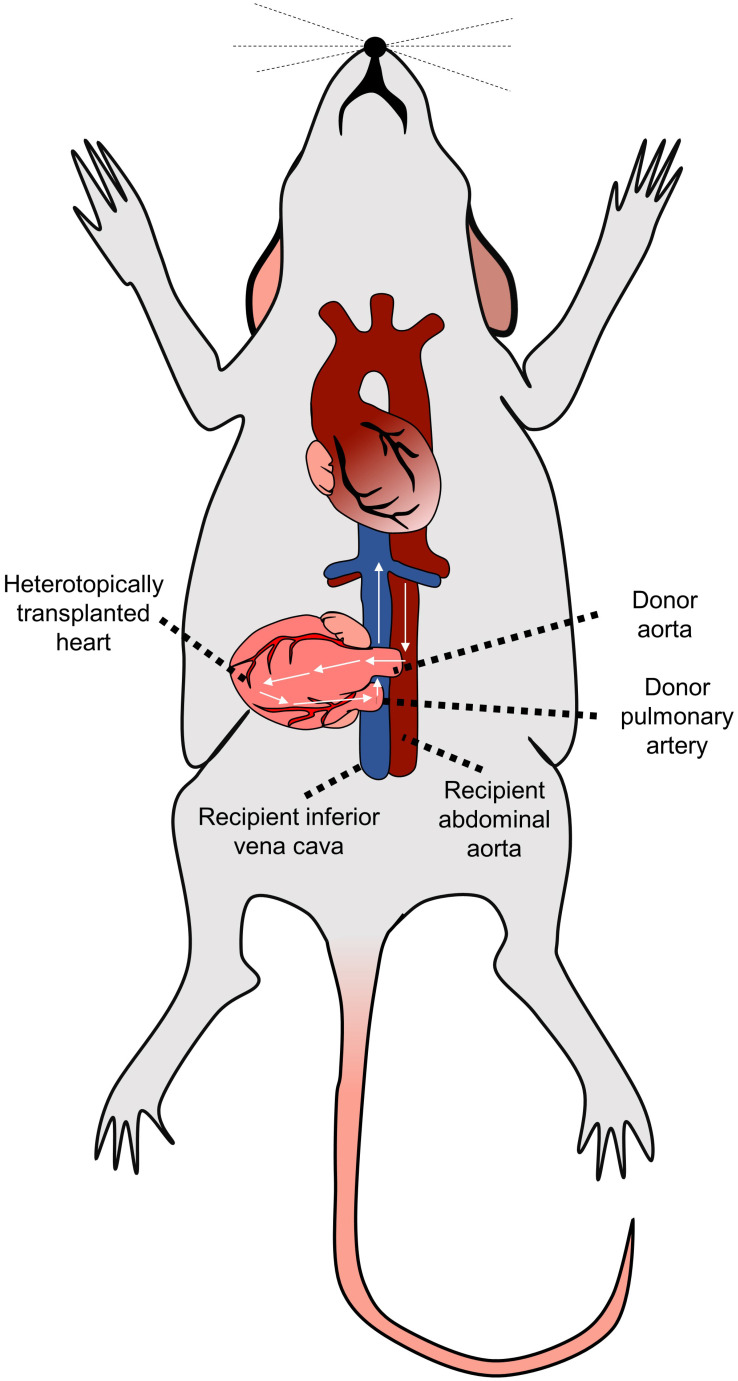
** Heterotopic heart transplantation model.** The donor's heart is located into the peritoneal cavity by connecting the recipient's abdominal aorta to the donor's ascending aorta and the recipient's inferior vena cava to the donor's pulmonary artery.

While in humans, donor's heart is transplanted orthotopically, i.e., into the location of the original recipient's heart, the most often used murine model of cardiac transplantation is based on heterotopic heart transplantation, as the small size of mice generally prohibits the successful performance of fully functional orthotopic heart transplantation (OHT) 46. The heterotopic heart transplantation model was first described in 1973 by Corry et al. 48. Within this model, the heart graft is transplanted into the recipient's peritoneal cavity by connecting the recipient's abdominal aorta to the donor's ascending aorta and the recipient's inferior vena cava to the donor's pulmonary artery 48,49 (Figure 2). This setting results in good coronary perfusion; however, the donor's heart's workload is minimal. The graft itself is non-functional - this gradually leads to the atrophy of the myocardium and formation of the mural thrombi in the left ventricle in the post-transplantation period.

The described model allows us to study alloreactivity associated with acute rejection. One of the most significant advantages of the model is the easily accessible monitoring of graft vitality/rejection just by palpating the allograft's heartbeats. On the other hand, the study of the vasculopathy is limited as characteristic proliferative neointimal lesions associated with chronic allograft vasculopathy in humans are more prone to develop in the arteries located in the epicardial fat 50 while in the mice, there is a low number of such epicardial arteries (only short segments of coronary arteries located near the aortic root are running epicardially) 51.

### miRNAs altered in murine models of cardiac allograft transplantation

#### microRNA profiling experiments revealed several miRNAs to be associated with ischemia-reperfusion injury and graft rejection

One of the first attempts to determine the role of miRNAs in the setting of heart transplantation was performed by Wei et al. in 2012 using murine syngeneic and allogenic heterotopic heart transplantation models 52. They identified the set of 74 miRNAs to be differentially expressed between the heart tissue of syngeneic and allogenic hearts and another 44 miRNAs differentially expressed in the graft infiltrating lymphocytes (GILs). Out of these miRNAs, 16 miRNAs were differentially expressed both in the heart tissue and GILs (miR-7a, -15b, -18a, -20a, -25, -92a, -130b, -142-3p, -148, -155, -182, -183, -200c, -361, -467a were upregulated and miR-434-3p was downregulated), out of which upregulation of miR-7a, -155, -182, and -183 and downregulation of miR-434-3p were confirmed by qPCR. The very same results, i.e., upregulation of miR-7, miR-155, miR-182, and miR-182 and downregulation of miR-434-3p in GILs was observed in the independent study by Huang et al. 53. The biological function of miR-182/183 was further investigated, and their expression was shown to increase during T-cell proliferation and to be decreased using calcineurin inhibitor cyclosporine A, suggesting the importance of miR-182/183 in immune cells` response to allogenes. Last but not least, the expression of miR-182 was further shown to be increased in circulating (not only graft infiltrating) lymphocytes and even in the plasma of mice during ACR, which provided one of the first signals indicating the potential use of circulating miRNAs for ACR non-invasive monitoring. In their subsequent study, Wei et al. identified CD4+ T-cells as the main cellular source of miR-182 in the cardiac allografts, and authors further showed that decrease in miR-182 levels induced by CTLA4 antibody, or in the general absence of miR-182 in knock-out animals, improves allografts survival 54.

Later on, another profiling experiment was performed by van Aelst et al. Their group focused not only on murine models but also on human ACR samples, and they aimed to identify potential common miRNA rejection patterns by also studying renal ACR 55. They identified 9 miRNAs to be increased (miR-21, -142-3p, -142-5p, -146a, -146b, -155, -222, -223, and -494) and miR-149-5p to be decreased in EMB from both mice and human myocardial samples with ACR. Out of these, miR-142-3p, miR-142-5p, miR-146b, miR-155, miR-223 were also differentially expressed in renal allografts. In a murine model of cardiac transplantation, both miR-155 knockdown and pharmacological inhibition using antagomir-155 caused ACR attenuation - this may be partially due to SPI1 miR-155-mediated inhibition (SPI1 is an important actor in the IL-6 pathway).

Zhou et al. in 2013 also performed a miRNA profiling study 56. However, the authors focused not on the changes induced by the allogeneic immune response but on the changes caused by I/R injury. In the syngeneic heart transplantation model, they used BALB/C mice and created the I/R group by inducing 18-hour-long cold ischemia. They further compared myocardial miRNAs expression with the non-I/R group, in which they omitted cold ischemia and transplanted the hearts immediately from the donor to the recipient. The expression of 20 miRNAs was up-regulated while the expression of 39 miRNAs was downregulated; out of these, increased expression of 10 miRNAs (miR-328, -346, -705, -711, -714, -744, -1892, -2137, -5130, and -5099) and decreased expression of another ten miRNAs (miR-24, -49, -128, -181, -210, -328, -362, -423, -490 and -532) were confirmed by qRT-PCR. Similar profiles were further observed in hypoxia/reperfusion-treated primary cardiomyocytes. Subsequent gene expression analysis revealed 48 genes and 18 signaling pathways to be affected by I/R injury 56.

Comparing miRNA profiling results between ACR and I/R injury, only a few individual miRNA overlaps (specifically miR-181 and miR-744) 54,56,57. This demonstrates that different miRNAs are involved in regulating distinct processes after CAT and further underlines their importance as potential biomarkers in the field.

#### miR-155 is a promising theranostic target associated with leukocytes function, and its levels are up-regulated during both acute rejection and vasculopathy development

In all the works described above, one of the miRNAs was repetitively confirmed to be up-regulated during acute rejection: miR-155. Due to its significant role in regulating immune response 58, many authors focused on studying its CAT settings role. Feng et al. demonstrated that miR-155 expression gradually increases in GILs and in lymphocytes isolated from spleen and peripheral lymphocytes over time in the post-transplantation period 59. GCSK3β, a significant member of the serine/threonine kinase GSK-3 family, was confirmed as a mechanistic target of miR-155, and its downregulation may support lymphocyte proliferation 59. Interestingly, in another study, miR-155 expression was observed to be downregulated by intravenous and intrathymic injection of mesenchymal stem cells, which resulted in suppressing T-cell response and prolonged cardiac allograft survival 53. The miR-155 expression is known to be induced as soon as 48 h after CD4+ T-cell activation, which makes it a very promising biomarker for early ACR detection 60. Increased expression of miR-155, together with increased expression of miR-146a and decreased expression of miR-451, was observed not only in the allogeneic transplantation but also in the model of xenogeneic (rat-to-mouse) transplantation - Li et al. performed microarray analysis and identified 24 and 25 miRNAs to be differentially expressed between xenografts and allografts 24 a 40 h after transplantation, respectively 61. Finally, to underline miR-155 relevance for ACR diagnostics, a recent meta-analysis focusing on miR-155 in solid organ transplantation showed that miR-155 was increased in 275 transplanted patients, including not only the heart but also renal and lung transplantations 62. Altogether these data suggest that increase in miR-155 levels is a common sign of acute rejection response related to the activity of CD4+ T-cells.

Furthermore, Gao et al. showed that increasing miR-155 expression is related not only to the activity and function of CD4+ T-cells but also to the function of dendritic cells (DC) 63. miR-155 expression increases even during DC differentiation and promotes their maturation, IL-12 production, and increases their capability to stimulate allogeneic T cell proliferation. The mechanistic target involved in mentioned processes was identified as SOCS1, and the application of antagomiR-155 in the murine allograft transplantation model altered its levels and protected cardiac allografts from rejection 63.

Besides the roles of miR-155 in the ACR process, miR-155 seems to be also involved in the graft vasculopathy development - Zhang et al. used miR-155 knock-out mice and found out that animals lacking miR-155 are partially resistant to allograft rejection, and that application of recombinant IL-17A removes this protective effect. All in all, the authors suggest that miR-155 regulates immune response in chronic cardiac transplantation and represents a diagnostic and potentially therapeutic target 64. To further support the potential modulation of miR-155 as a therapeutic target, in their recent study Yi et al. showed that miR-155 antagomir might be administered packed within the cationic microbubbles 65. After these bubbles were injected, using ultrasound targeted microbubbles destruction, miR-155 antagomir was explicitly delivered to cardiac tissue, thus preventing the potential off-site effect of systemic administration. Specifically, in Yi et al. study, such delivery of miR-155 antagomir prolonged cardiac allograft survival and decreased both the expression of inflammatory cytokines and the extent of inflammation infiltration of the myocardium 65.

#### miR-21 is up-regulated during ACR and promotes fibrotic changes in cardiac allografts

Both above-discussed miRNAs (miR-155 and miR-182) were studied, especially in ACR-related immune response and leukocyte function settings. Another miRNA repeatedly identified in the profiling experiments of EMB was miR-21, and its role in the murine model of cardiac allograft transplantation was studied by Gupta et al. 66. Authors initially performed miRNA profiling of murine allo- and isografts and identified miR-21, -142-3p, -146a, -155, -210 and -1224 to be increased (above two-fold) and miR-1, -29c, -30a, -30a*, -30c, -30d, -30e, -99a, -100, -126-3, -133a, -133b, -149, -185, and -208a to be decreased. Their further work showed that the level of miR-21 strongly correlated with fibrosis of cardiac allografts, and locked nucleic acid against miR-21 could reverse the fibrosis. miR-21 was shown to be part of the IL-6 axis and involved in the monocyte-to-fibrocyte transition. This happens by miR-21 activation of CCL2 via AP-1, leading to activation of the fibrotic gene program. Interestingly, similar findings that inhibition of miR-21 reduced fibrosis was also described for kidney allografts 67.

#### miR-142 as a crucial regulator of allograft survival via T-cell specific mechanisms

miR-142 was altered in murine experiments during ACR in the experiments by Wei et al. and Van Aelst et al. 52,55. It was also studied in several human studies by the team of Sukma-Dewi as a potential ACR biomarker (as discussed in 4.2.1) 68-70. In a recent survey of Anandagoda et al., miR-142 was also the main focus, and on several murine models, authors showed that miR-142 is a crucial mediator of both ACR and AMR 71. Authors first showed and confirmed that both variants of miR-142 (i.e., miR-142-3p and miR-142-5p) are increased in cardiac allografts on days 4 and 8 after the operation and that survival of such allografts is below ten days. Authors then created miR-142 knock-out C75BL/6 mice (*MiR142*^-/-^) and showed that cardiac allografts from BALB/C mice showed indefinitely (over 100 days) survival without the need of any other immunosuppression. This was not the cause of vice versa transplantation, i.e., using *MiR142^-/-^* hearts as donors into BALB/C recipients resulted in ACR/AMR as observed in wild-type animals suggesting that miR-142 is involved in the immune reaction on the recipient's site. Searching further for the mechanisms underlying their results, they created a model with T-cell conditional deletion of miR-142. Even in such a model, the donor's hearts continued to beat for more than 100 days, pointing out the importance of miR-142 in T-cells response to the alloantigens. Specifically, in all models of miR-142 deficiency, an increase in Treg cells subpopulation was observed 71.

#### Other miRNAs whose function was studied in the murine models of cardiac allotransplantation

An overview of other miRNAs studied in the context of cardiac allograft rejection in murine models is provided in Table 2. Briefly, miR-146a 72 and miR-744 57 are involved in regulating Treg cell function. Decreasing miR-146a levels or increasing miR-744 levels promote the inhibitory/immunomodulatory function of Treg cells, which prolongs the survival of cardiac allografts. miR-499 73 and miR-669b-3p 74 levels were detected to be increased during ACR, and this increase is associated with various immune cell functions. Changes in miR-499 associated with the metabolic activity and alterations in miR-669b-3p levels affected proliferation and apoptosis of CD4+ T-cells. Inhibition of expression of both miRNAs improved allografts survival.

Lastly, miRNAs levels were also shown to be altered by immunosuppressants or to be necessary for their cellular effects. Specifically, miR-377 was shown to mediate cyclosporine A-induced apoptosis 75, while changes in the expression of a group of other miRNAs were associated with the administration of eicosapentaenoic acid 76.

**Figure 3:**
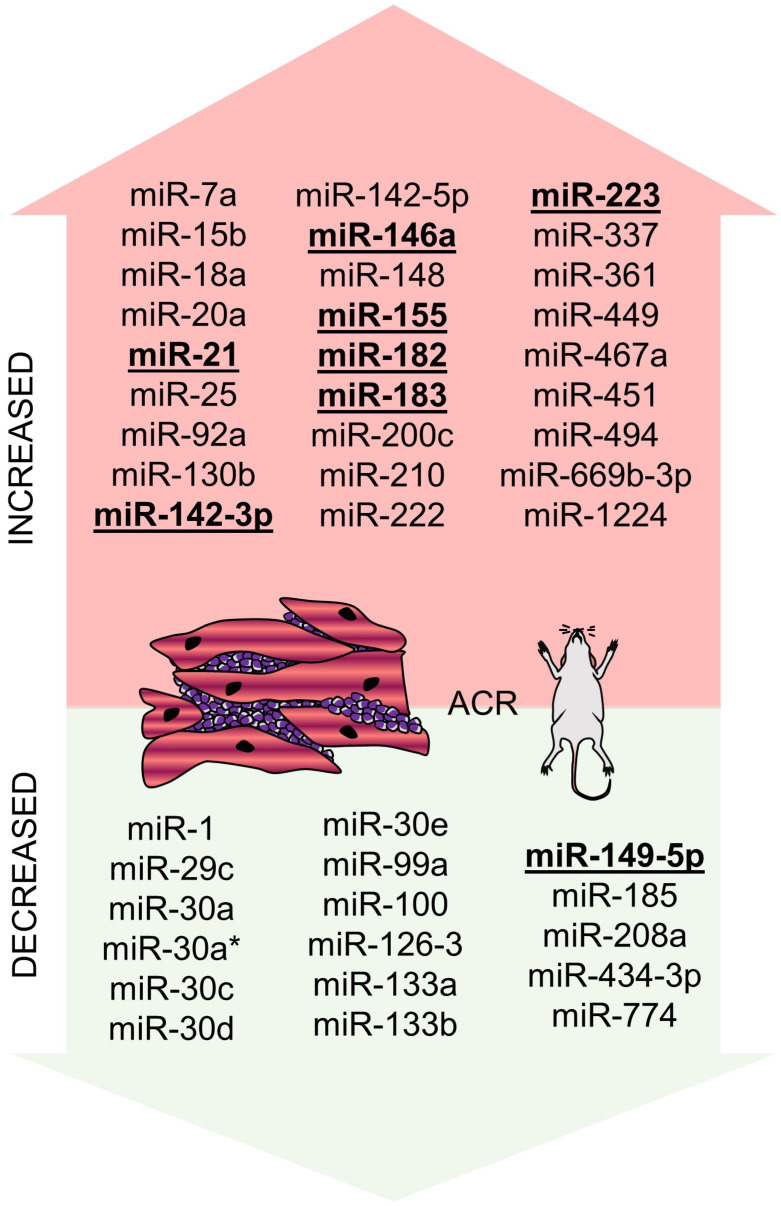
** miRNAs altered in ACR identified in the murine experiment.** miRNAs altered in ACR identified in the murine experiment - increased/overexpressed miRNAs are shown in the upper part of the figure, decreased/underexpressed miRNAs are shown in the lower part of the figure. miRNAs reported in at least two studies are marked in **bold** and underlined. **Abbreviations**: ACR: acute cellular rejection.

Overall summary of miRNAs altered during ACR in murine models is provided in Figure 3.

## Tissue and circulating microRNAs altered in patients after heart transplantation

Previous text was focused on the studies performed using experimental models of heart allograft rejection. These studies provide us deep insight into the pathophysiology of allograft rejection. All in all, it shows that levels of various miRNAs are altered during allograft rejection and may thus hypothetically be used in clinical practice.

The studies performed in human subjects are limited in their size and higher variability caused by the individuality of all involved subjects, their medical history, used treatments, and concomitant complications (traumas, infections, etc.), that can be avoided in the laboratory settings but are unavoidable in the actual clinical practice. Nevertheless, such studies' value is priceless, as not all data from animals' models can be transferred to the clinical practice, and data from clinical trials represent the cornerstones to improve care for patients after OHT.

In general, in human studies, miRNAs may be determined in EMB or in the extracellular fluids. Due to miRNAs' high stability in tissues and extracellular fluid, even analysis of already archived paraffin-embedded EMBs or deeply frozen plasma/serum samples is possible, which provides the opportunity for retrospective studies on larger cohorts.

Most of the studies described further consist of the discovery phase, either performed using next-generation sequencing, microarray qPCR analysis, or *in silico* analysis/literature search and of the validation phase, where miRNAs identified in the discovery phase are validated using qRT-PCR. Commonly, the “sample trios” are used, i.e., including samples obtained before rejection, during rejection, and after rejection - this study design shall be optimal for identifying miRNAs whose levels either increase during the ACR and then decreased after the ACR or vice versa - this “rise and fall” or “decrease and rise” pattern shall reveal miRNAs influenced by ACR itself.

In the further text, we will firstly focus on data obtained from EMBs and extracellular fluid in the context of acute rejections and then on limited data focusing on graft vasculopathy.

### Several different miRNAs are altered in endomyocardial biopsies in patients after heart transplantation during acute rejection

In the settings of ACR (as also mentioned in the previous text), van Aelst et al., besides performing microarray analysis of murine samples, also tested human EMB samples and identified nine miRNAs to be increased (miR-21, -142-3p, -142-5p, -146a, -146b, -155, -222, -223, and -494) and miR-149-5p to be decreased both in mice and human. 55 Out of these miRNAs, increased miR-21 levels within EMBs of patients with ACR were further reported in the study by Gupta et al., and mechanistically, miR-21 was shown to be involved in the fibrotic processes 66.

**Figure 4:**
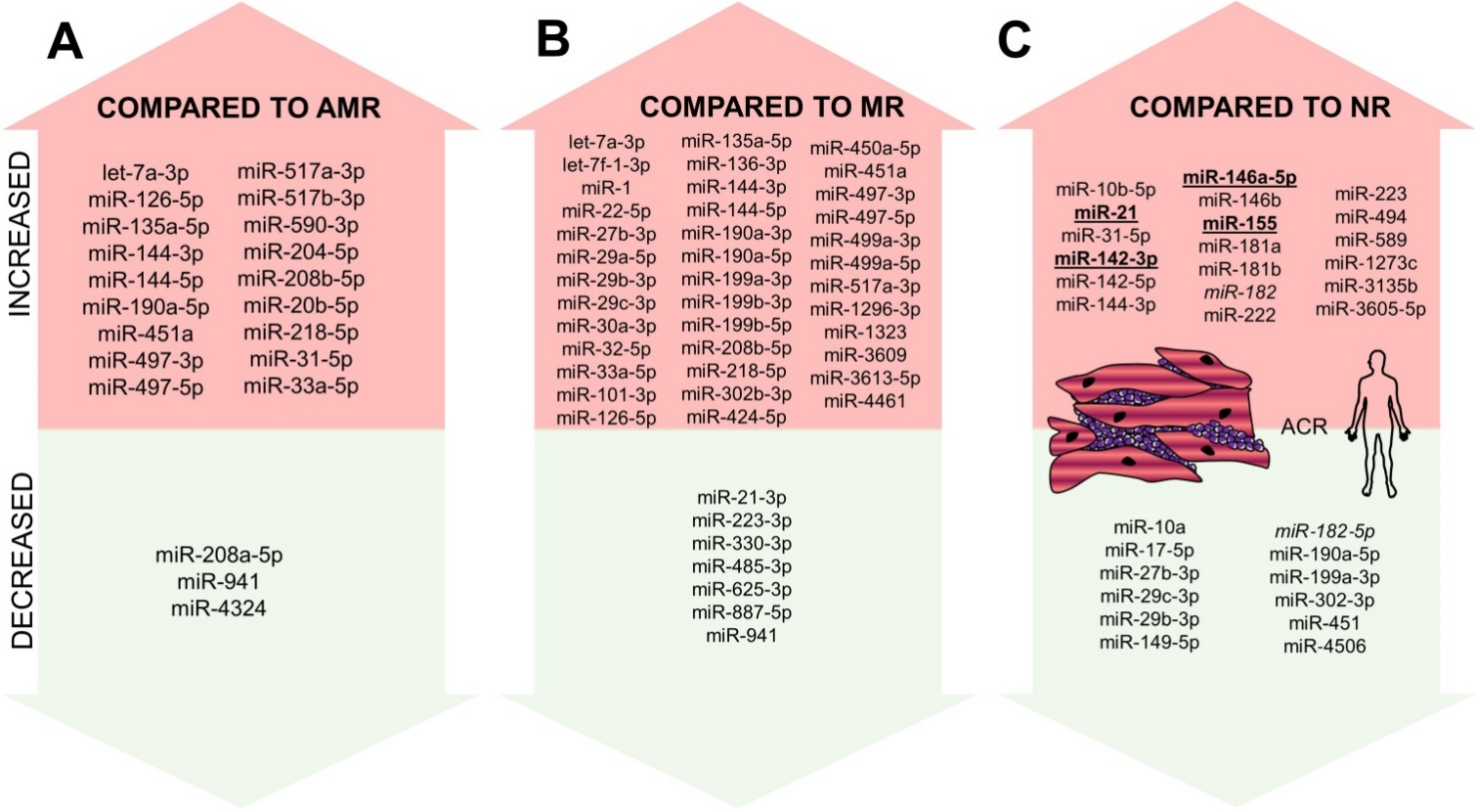
** miRNAs altered in ACR from EMBs identified in human samples.** miRNAs altered in ACR identified in the human experiment - increased/overexpressed miRNAs are shown in the upper part of the figure, decreased/underexpressed miRNAs are shown in the lower part of the figure. Part A: miRNA increased/decrease in ACR compared to AMR; Part B: miRNAs increased/decreased in ACR compared to MR; Part C: miRNAs increased/decreased in ACR compared to no-rejection samples. miRNAs reported in at least two studies are marked in **bold** and underlined. miR-182 is marked in *Italic* as it was reported to be both increased and decreased. **Abbreviations**: ACR: acute cellular rejection; AMR: antibody-mediated rejection; MR: mixed rejection; NR: no-rejection.

In addition to profiles obtained from microarrays, miRNA profiling was used in two recent studies. Di Francesco et al. included 33 patients after OHT and aimed to identify the differences between ACR, AMR, and MR 5. miRNA profiles of AMR and MR were similar (difference in just two miRNAs: miR-31-5p and miR-20b-5p), suggesting similar pathophysiological pathways to be activated in these two types of rejection. In comparison, 18 miRNAs were shown to be overexpressed, and three miRNAs under-expressed in the ACR compared to AMR and 39 miRNAs to be overexpressed and seven miRNAs to be under-expressed in the ACR compared to MR (Figure 4A-B). Out of these miRNAs, lower miR-31-5p, -218-5p and -144-3p expression was found in pAMR and MR compared with controls and lower levels of miR-29c-3p, -29b-3p, -199a-3p, -190a-5p, -27b-3p, and -302-3p were able to differentiate rejection in general compared to non-rejection samples. Using logistic regression modeling, combination of five miRNAs (miR-27b-3p, -29b-3p, -199a-3p, -208a-5p, and -302) was able to differentiate ACR from other rejection types, combination of three miRNAs (miR-208a-5p, -126-5p, and -135a-5p) was able to differentiate MR from the other types and lastly, combination of four miRNAs (miR-208a-5p, -29b-3p, -135a-5p, and -144-3p) was able to differentiate AMR from other rejections.

We also performed miRNA profiling of ACR EMB samples within our research group using next-generation sequencing with the focus put solely on ACR 77. Including 38 patients' samples trios, we identified 11 miRNAs to be altered during the ACR process: levels of six miRNAs increased during the ACR (hsa-miR-3135b, -146a-5p, -589-5p, -1273c, -31-5p, and -3605-5p), levels of three miRNAs decreased during the ACR (hsa-miR-182-5p, -17-5p and -4506) and levels of two miRNAs increased during ACR (as compared with samples before rejection); however, their increase further continued even after the rejection was over (hsa-miR-144-3p and hsa-miR-10b-5p). Out of these miRNAs identified within NGS, levels of miR-144, -589, and -182 were confirmed to be statistically significantly altered during the rejection on the independent cohort using qRT-PCR. Taking into account levels of all 11 miRNAs, we created an ACR score showing the specificity of 91% and sensitivity of 68% for determining the presence of ACR in the sample.

The apparent overlap of all the studies listed above is only in a few miRNAs (Figure 4C). This may be explained by different methodologies, different normalization methods, and also by the slightly different aim of individual studies as some of them were focusing on the comparison of particular rejection types 5 while the others were focused on the identification of ACR biomarker 77. Importantly, EMB samples with only grade 2R rejections were included in some studies, while grade 1R rejections were studied in the others 5,77. Such variability makes the generalization of results of EMB studies quite challenging; however, all of the performed studies point out the crucial fact that if the proper set of miRNAs is found, it can be used to distinguish the rejection sample from the non-rejection sample and even to distinguish individual rejection types. This may hypothetically remove the interobserver variability that is sometimes reported in the histopathological analysis of EMBs.

### Circulating microRNA as biomarkers of ACR

#### Circulating microRNA as biomarkers of ACR as revealed by microarray profiling

Determining miRNAs in EMBs still requires EMB performance and thus does not allow its avoidance or decrease in the number of performed EMBs, which would increase patients' quality of life (e.g., there will be shorter or no hospitalizations for EMB performance) and safety (especially by avoiding rare but severe complications of EMB, such as cardiac tamponade or ventricular fibrillation). Studies focusing on circulating miRNAs (summarized in Table 3) bring new hope for patients after OHT, that number of EMBs may be reduced, or even EMBs themselves may be replaced - the pathway to this change is currently filled with unexplained and unsolved matters; however, results of so far performed studies are promising.

The first proof-of-concept study to find circulating RNA profile typical for ACR was performed by Sukma-Dewi et al. in 2013 68. Their study included just a small group of ten patients undergoing their first histologically proven ACR. Using sample trios and qPCR-based array profiling, seven miRNAs (miR-142-3p, -101-3p, -424-5p, -27a-3p, -144-3p, -339-3p, and -326) have been identified to be differentially expressed during the ACR more than 1.5-fold. Out of these, miR-326 and miR-142-3p showed acceptable results of ROC analysis with the area under the curve (AUC) 0.86 and 0.80, respectively. These two miRNAs, together with miR-101, are known to be expressed by immune cells and to participate in the maintenance of self-tolerance. Thus, they may potentially reflect even the immunological changes occurring during the ACR. As the initial cohort was indeed small, authors validated their previously identified set of seven miRNA on a larger independent cohort - they compared samples from patients with rejection (n = 26) and without rejection (n = 37) collected at the Prevention of Organ Failure (PROOF) Centre of Excellence (Vancouver, Canada). ACR was defined as ≥ 1R according to ISHLT 2004 classification, and control samples from patients without rejection were matched to ACR subjects according to post-transplant sample collection time, age, and sex 69. In this independent cohort, miR-142-3p and miR-101-3p had the best diagnostic performance with AUC 0.78 and 0.75, respectively. Importantly, these two miRNAs' levels were not correlated with CRP and serum creatinine levels, suggesting they are not reflecting the general inflammation status and that they are not affected by kidney function. Their levels were also independent of calcineurin inhibitor levels 69.

Further deepening the knowledge about the discovered miRNAs, in the subsequent study, the authors focused on miR-142-3p, miR-92a-3p, miR-339-3p, and miR-21-5p and showed that their levels are explicitly increased in exosomes in patients with ACR, and authors further provided the evidence that miR-142-3p originates from the activated T-cells from which it is transferred into endothelial cells (thus acting as a paracrine mediator). In endothelia, miR-142-3p increases vascular permeability and partly mediates allograft damage and destruction 70. The uptake of miR-142-3p into endothelial cells explains its presence in the allografts, and importantly, its increase was also detected in the kidney 78 and liver 79 transplants. Together with the results of the recent study by Anandagoda et al. (as discussed in 3.2.4) 71 this raises a potential question whether miR-142-3p may represent a potential universal marker of rejection released to the systemic circulation from T-cells and whether modulating its levels may protect allografts from rejection. This needs to be validated by further studies.

Besides the three studies performed by Sukma-Dewi et al., another miRNA profiling using qPCR microarrays was recently performed by Constanso-Conde et al. 80. The authors choose a standard two-step approach and performed a discovery and validation phase. In their discovery phase, the authors included 21 sample trios and identified 148 altered miRNAs in transplanted patients' serum samples. After comparing samples before and during rejection, four miRNAs (miR-181a-5p, -339-3p, let-7f-5p, and -505-3p) showed to be differentially expressed. Still, only miR-181a-5p differed when comparing samples during rejection and after rejection, thus establishing a “rise and fall” pattern suggesting its involvement in the rejection process. On the validation cohort including 45 ACR and 45 control samples, miR-181a-5p was confirmed to be increased in ACR with an AUC 0.804 (95% CI: 0.707-0.880), showing sensitivity and specificity of 78% and 76%, respectively.

#### Circulating microRNA as biomarkers of ACR as revealed by *in silico* analysis and literature search

Further studies focusing on circulating miRNAs as biomarkers of ACR were performed using *in silico* analysis. In 2014, van Huyen et al. conducted an extensive study including 113 patients after heart transplantation 81. Within their *in silico* analysis, they preselected 14 miRNAs, based on databases screen and their known biological roles and divided them into three categories: i) miRNAs expressed in endothelium and related to endothelial activation (miR-92a, -126, -221, -296); (ii) miRNAs expressed in cardiomyocytes and related to myocardial remodeling and function (miR-21, -31, -208) and (iii) miRNAs related to leukocytes functions and inflammatory status (miR-10a, -142-3p, -155, -181a, -181b, -182, and -451). Within the EMBs, in the test cohort of 60 patients, seven miRNAs (miR-10a, -21, -31, -92a, -142-3p, -155, and -451) were shown to be differentially expressed between individuals with histologically proven ACR and the set of 30 matched control patients after heart transplantation without ACR (matching included recipient and donor ages, time of cold ischemia and time from transplantation to index biopsy as well as the immunosuppressive regime). Out of these seven miRNAs, four of them were differentially expressed in serum samples: miR-10a showed decreased expression, and the other miRNAs (miR-31, -92a, -155) showed increased expression. These results were then validated on an independent cohort of 53 individuals with ACR collected from three various French transplantation centers, and AUC for each miRNA remained high: miR-10a had AUC 0.981, miR-31 had AUC 0.867, miR-92a had AUC 0.959, and miR-155 had AUC 0.974.

Guo et al. chose a different strategy and focused on one specific miRNA, namely miR-29a 82. miR-29 is associated with cardiomyocyte injury, and Guo et al. determined its blood sample levels from 50 healthy individuals and more than 500 patients after heart transplant with R0, R1, and R2/3 grades of rejection. They observed that serum miR-29 levels were gradually increasing comparing healthy controls (47.6 ± 28.4 copies/μl), R0 group (100.8 ± 42.4 copies/μl), R1 group (537.5 ± 84.3 copies/μl) and R2/3 group (1478.4 ± 198.7 copies/μl). This study's levels of miR-29 correlated with numerous biochemical parameters, including cTNI, NTproBNP, white blood cells count, and creatinine levels, but not CRP levels and patients' age. Interestingly, miR-29 levels were also determined in various time points after heart transplantation, and its levels gradually decreased over time; miR-29 levels were 1963.5 ± 214.73 copies/μl after six months, 1242.5 ± 103.8 copies/μl after a year, 825.6 ± 58.2 copies/μl after two years, 413.8 ± 61.9 copies/μl after three years and 270.6 ± 34.6 copies/μl after four years from transplantation. These results probably reflect the decreasing cardiomyocyte injury of the heart allografts over time as the donor's immune system accepts the allograft. In the multivariate analysis, miR-29 levels predicted 2R/3R grade of rejection with high sensitivity and specificity (AUC 0.79 (0.72-0.86) with the sensitivity of 79.6% and specificity of 53.8%).

Last but not least, in the very tiny study from Iran, nine patients after OHT and three patients indicated for coronary-artery bypass grafting were included, and levels of previously identified miRNAs associated with graft rejection (miR-133, -155, and -326), together with troponin were determined 83. Due to the small study sample size, only statistically non-significant trends in miRNA levels were observed: miR-155 and miR-326 levels tended to increase while levels of miR-133 tended to decrease in patients who deceased (n = 3) within one month after OHT, compared to those who survived (n = 6) longer than one month after OHT. On the other hand, increased troponin levels showed high sensitivity and specificity with AUC 0.98 (95% CI 0.93-1.00) in discriminating these two groups. Needless to point out that this study bears several methodological issues besides the small sample size, another one being the fact that no EMB was performed, as the insurance companies in Iran do not cover them; thus, no information on the actual rejection status of both survivors and non-survivors is available.

#### Circulating miRNAs as early markers of cardiac damage in the first weeks after transplantation

Besides the studies focusing on ACR monitoring as described above, in 2013, Wang et al. performed a small-sized study, including seven patients after OHT, focusing not on ACR monitoring but on monitoring of cardiac damage in the first two weeks after OHT 84. Authors selected cardio-specific miRNAs that were previously reported to be increased in plasma/sera of patients after myocardial infarction, specifically miR-133a, miR-133b, and miR-208a, and determined their levels on day 0 (immediately after the operation), and then on days 1, 2, 3, 7 and 14. The levels of all of the miRNAs were significantly increased after the surgery and gradually decreased. Especially miR-133b showed significant correlation with troponin and other clinical parameters, such as ventilation time and length of ICU stay, thus also predicting patients' recovery after heart transplantation 84, however, due to limited study size, a generalization of results is not possible.

### Tissue and circulating miRNAs as biomarkers of cardiac allograft vasculopathy

Long-term outcomes of patients after OHT are significantly affected by the development of CAV, which is usually diagnosed by coronary angiography, intravascular ultrasound, or OCT. CAV is characterized by the development of concentric fibromuscular intimal hyperplasia lesions within epicardial and intramyocardial arteries and by developing eccentric atherosclerotic plaques in the epicardial arteries 11.

In 2015, Singh et al. included 52 patients undergoing SCG in the interval from 5 to 15 years after OHT and found out that levels of miR-92a-3p were 1.87-fold (p < 0.05) higher in patients with CAV than in patients indicated for coronary angiography for coronary artery disease without CAV. They also examined the levels of other endothelium-related miRNAs (miR-21-5p, -92a-1-5p, -126-3p, and -126-5p); however, observed changes were not statistically significant 85. In 2017, Neumann et al. performed miRNA profiling of vasculopathy patients 86. Their microarray analysis identified five candidate miRNAs: four of them were up-regulated (miR-98, -155, -204, -628-5p), while one was downregulated (miR-34a) in patients with CAV compared to those with no signs of CAV. After qRT-PCR validation, only miR-628-5p and miR-155 remained potential biomarkers for CAV with a sensitivity of 72.22% and a specificity of 83.33%, and sensitivity of 88.89% and specificity of 61.11%, respectively 86.

In addition to the results mentioned above focusing on circulating miRNAs, in a recent study by Heggermont et al., authors determined levels of endothelium-enriched miR-126-3p in EMBs from patients with and without CAV and showed decreased expression of this miRNA in individuals with CAV 87. ROC analysis showed an AUC of 0.786, a sensitivity of 76%, and a specificity of 73%. This study is of clinical importance as a biomarker of CAV from EMB is not available and determining CAV from EMB without the need for coronary angiography or OCT may enable better CAV surveillance just by additional analyses of already obtained EMBs used for ACR diagnostics. However, whether the expression of miR-126-3p in EMBs will help diagnose CAV at an early stage or will enable CAV surveillance still needs to be verified on larger patients' groups.

## Conclusions and future directions

Studies focusing on biological roles of miRNAs after CAT clearly show their involvement in the regulation of the function of T-cells (including activation, energetic metabolism, response to antigen, etc.), dendritic cells (affecting their ability to present antigens and their maturation), endothelial cells (affecting their function and permeability), cardiomyocytes (affecting, e.g., their function and apoptosis) and potentially all other cell types involved in the complex process of cardiac allograft tolerance and rejection, including ACR, AMR, MR or CAV. Animal models of CAT enable us to “dissect” this complex process into individual subprocesses and thus to identify potential clinically relevant targets with translational potential - application of both miRNAs mimics or inhibitors and their delivery into the transplanted heart in animal models lead to amelioration of the rejection process and resulted in better allograft survival and function. Despite no data being available for such miRNA-based therapeutics in the CAT field for humans, clinical trial Phase Ib (ClinicalTrials.gov Identifier: NCT04045405) has been recently finished. Within this study, an anti-miR-132 oligonucleotide (CDR132L) was used to treat patients with heart failure of ischemic origin 88. This therapy was safe and well-tolerated and resulted in a decrease in NTproBNP levels and also in a decline of levels of several markers of cardiac fibrosis, suggesting positive effects on the course of heart failure in treated individuals 89. It is thrilling that miRNA-based therapeutics are starting to be tested in patients with CV diseases, and future development of novel treatments even for patients after OHT may be expected.

Furthermore, besides their therapeutic potential, circulating miRNAs represent the clinically valuable group of biomarkers. Recently, the very first clinically validated diagnostic kit has started to be used for the non-invasive diagnostics/screening of gastric cancer using levels of 12 plasmatic miRNAs 90. In the cardiovascular field, the discovery of clinically reliable markers still needs to solve numerous technological challenges; especially, there is the need to unify both the preanalytical (e.g., samples collection, extracellular fluid selection, storage, and handling) and the analytical (microarray analysis and next-generation sequencing standardization, the unification of internal or spike-in control use to normalize obtained qRT-PCR data) processes. This lack of unification makes comparison and generalization of results from individual studies challenging and sometimes even impossible. Moreover, in the CAT field, another problem needs to be solved as the currently most often used model of ACR biomarker search using sample trios (i.e., using samples before, during, and after *histologically* verified rejection) may have its pitfalls, as *miRNA levels* may hypothetically be altered even before the *histological* changes occur, thus skewing the subsequent analyses in trying to find out “rise and fall” or “fall and rise” patterns. However, and generally speaking, it is more than evident that determining several miRNAs from one sample will be necessary to create a diagnostic or prognostic score providing high enough sensitivity and specificity to diagnose either ACR or CAV from the so-called “liquid biopsies” (i.e., from plasmatic or serum samples).

Future research in the field of CAT shall focus on identifying novel and more rejection-specific miRNAs as well as on validation of already identified miRNAs on larger and independent cohorts. Studies determining the miRNA levels in one patient during more time points after OHT are also needed to reveal dynamics of circulating miRNA levels after OHT and decide on their prognostic role in predicting ACR, AMR, MR, or CAV development. In the studies described above, only a little attention has been paid to AMR, as its occurrence in the studied cohorts was minimal. Thus, studies focusing on this rarer phenomenon are also emphasized.

All in all, miRNAs represent intriguing tiny molecules with enormous potential in the field of cardiac allograft transplantation theranostics. They may improve our understanding of the individual rejection processes, which may lead to the development of novel therapies or diagnostic/prognostic stratification tools. The use of such tools may then result in personalizing our care for patients after OHT, increasing both patients' safety and quality of life.

## Figures and Tables

**Table 1:** Commonly used databases to predict miRNA targets and functions

Name	URL	Ref
TargetScan	http://www.targetscan.org/vert_72/	91
DIANA-TarBase-v8	https://carolina.imis.athena-innovation.gr/diana_tools/web/index.php?r=tarbasev8%2Findex	92
DIANA-microT-CDS	http://diana.imis.athena-innovation.gr/DianaTools/index.php?r=microT_CDS/index	93
miRTarBase	http://mirtarbase.cuhk.edu.cn/php/index.php	94
miRWalk	http://mirwalk.umm.uni-heidelberg.de/	95
miRecords	http://c1.accurascience.com/miRecords/	96
miRmap	https://mirmap.ezlab.org/	97
miRDB	http://www.mirdb.org/	98

**Table 2:** Other miRNAs associated with cardiac allografts rejection and function

miRNA	Description	Ref.
miR-146a	**Model:** BALB/c to C57BL/6J (B6) C57BL/6J recipients were injected with miR-146a antagomir prior allograft transplantation. miR-146a antagomir increased the T_reg_ proportion in splenocytes and blood cells, which promoted the survival of the donor's heart.	72
miR-449	**Model:** BALB/c to C57BL/6 miR-449a expression increase in PBMCs and GILs during allograft rejection and CD4^+^ T-cell activation. Inhibition of miR-449a caused alteration in metabolic potential (glycolysis and maximum glycolytic capacity decreased after miR-499 inhibition), decrease in mitochondrial respiration, and thus seems to be involved in regulating energetic metabolism of CD4+ T-cells.	73
miR-669b-3p	**Model:** C57BL/6 to BALB/c After initial miRNA profiling and identification of 93 up-regulated and 78 downregulated miRNAs, miR-669b-3p upregulation was confirmed by qPCR and was further studied using cell cultures. Indoleamine-2,3-dioxygenase (IDO) was determined *in silico* as a mechanistic target and using mouse 3 T3 cell lines overexpressing or knock-out in miR-669b-3p, levels of IDO were shown to be decreased or increased, respectively. Increased levels of IDO then inhibited CD4^+^ T cell proliferation and promoted apoptosis.	74
miR-774	**Model:** C57BL/6 to BALB/c Expression of miR-744 in CD4+CD25+T_reg_ cells from CAT mice was significantly lower. Use of agomir for miR-744 increased levels of miR-744 in CD4+CD25+T_reg_ cells in the CAT group but not in the control group. TNFRSF4 gene was identified as the miR-744 mechanistic target. Functionally, the inhibitory function of T_reg_ cells was significantly enhanced in miR-744 agomir treated mice, and this resulted in prolonged survival time of cardiac allograft.	57
miR-377	**Model:** Neonatal rat (0-3 d) primary cardiomyocytes Cyclosporine A (CsA) increased apoptosis of neonatal rat cardiomyocytes. Using microarray assay, 17 miRNAs were shown to be altered in CsA treated groups, out of which miR-377 was the most significantly increased. Transfection of miR-377 increased cardiomyocytes apoptosis and vice versa; anti-miR-377 decreased cardiomyocytes apoptosis even after the CsA treatment. XIAP and NRP2 were identified as miR-377 mechanistic targets.	75
miR-223	**Model:** C57BL/10 (H2-Kb) to CBA/N (H2-Kk) Transplanted animals were administered with eicosapentaenoic acid (EPA), which significantly prolonged the survival of cardiac allografts. Authors focused on the groups of 10 miRNAs previously reported by their group to be altered in hepatic allografts and confirmed that levels of miR-223 were increased in the rejection group while the levels of other miRNAs (mir-146a, -15b, -23a, -34a, -451, -101a, -101b, and -148a) were increased in animals treated with EPA, suggesting the role of miR-223 in allograft rejection and the role for the rest of miRNAs in the allograft tolerance.	76

**Table 3:** Circulating miRNAs altered during ACR, AMR and MR in extracellular fluid

Authors	Cohort size	Method	Source	ACR definition	Normalization	Increased in ACR	Decreased in ACR
68	D: 10 trios V: 10 trios	D: microarray V: qRT-PCR	Serum	≥ 2 (ISHLT 1990)	D: Global mean V: Reference gene (miR-451)	miR-27a, miR-101, miR-142-3p, miR-144, miR-326, miR-339-3p, miR-424	no referred
81	D: 30 AR; 30 NR V: 31 AR; 22 NR	D: *in silico* prediction V: qRT-PCR	Serum	≥ 1 (ISHLT 2004)	Reference gene (RNU48)	miR-31, miR-92a, miR-155	miR-10a
70	D: 5 ACR; 5 NR	D: microarray	Serum exosomes	≥ 2R (ISHLT 2004)	Reference gene (miR-451)	miR-21-5p, miR-92a-3p, miR-142-3p, miR-339-3p	no referred
69	V: 26 ACR; 37 NR	V: qRT-PCR	Serum	≥ 1 (ISHLT 2004)	V: UniSp6 spike-in	miR-27a, miR-101, miR-142-3p, miR-144, miR-326, miR-339-3p, miR-424	no referred
82	V: 275 ACR; 231 NR; 50 healthy	D: literature screen V: qRT-PCR	Venous blood	≥ 2R (ISHLT 2004)	Reference gene (RNU6)	miR-29	no referred
80	D: 21 trios; V: 45 ACR; 45 NR	D: NGS V: qRT-PCR	Serum	≥ 2R (ISHLT 2004)	Global Mean and miR-23b-3p + miR-30c-5p geometric mean	miR-181a-5p	no referred

Table summarizing individual studies focused on finding circulating miRNAs in patients after heart transplantation associated with acute rejections. **Abbreviations:** D: discovery phase; V: validation phase; AR: acute rejection (includes ACR + AMR); ACR: acute cellular rejection; AMR: antibody-mediated rejection; NR: no rejection.
